# Assessment of UV radiation effects on airborne mucormycetes and bacterial populations in a hospital environment

**DOI:** 10.1038/s41598-024-53100-5

**Published:** 2024-02-01

**Authors:** Mohammadmahdi Sarkhoshkalat, Mahdi Ahmadi Nasab, Mohammad Reza Yari, Seyed Saeed Tabatabaee, Vahid Ghavami, Fatemeh Joulaei, Maryam Sarkhosh

**Affiliations:** 1 Department of Mechanical Engineering, Islamic Azad University, Mashhad, Iran; 2 Student Research Committee, Department of Environmental Health Engineering, School of Health, Mashhad University of Medical Sciences, Mashhad, Iran; 3 Social Determinants of Health Research Center, Mashhad University of Medical Sciences, Mashhad, Iran; 4 Department of Management Sciences and Health Economics, School of Health, Mashhad University of Medical Sciences, Mashhad, Iran; 5 Department of Biostatistics, School of Health, Mashhad University of Medical Sciences, Mashhad, Iran; 6 Department of Environmental Health Engineering, School of Health, Mashhad University of Medical Sciences, Mashhad, Iran

**Keywords:** Infectious diseases, Health services

## Abstract

Infections, such as mucormycosis, often result from inhaling sporangiospore present in the environment. Surprisingly, the extent of airborne Mucormycetes sporangiospore concentrations remains inadequately explored. This study aimed to assess the influence of UV radiation on microbial populations and Mucormycetes spore levels within a hospital environment in northern Iran. A comprehensive dataset comprising 298 air samples collected from both indoor and outdoor settings was compiled. The culture was conducted using Blood Agar and Dichloran Rose Bengal Chloramphenicol (DRBC) culture media, with Chloramphenicol included for fungal agents and Blood Agar for bacterial. Before UV treatment, the average count of Mucormycetes ranged from 0 to 26.4 ± 25.28 CFU m^−3^, fungal agents from 2.24 ± 3.22 to 117.24 ± 27.6 CFU m^−3^, and bacterial agents from 29.03 ± 9.9 to 359.37 ± 68.50 CFU m^−3^. Following UV irradiation, the averages were as follows: Mucormycetes ranged from 0 to 7.85 ± 6.8 CFU m^−3^, fungal agents from 16.58 ± 4.79 to 154.98 ± 28.35 CFU m^−3^, and bacterial agents from 0.38 ± 0.65 to 43.92 ± 6.50 CFU m^−3^. This study, notably marks the pioneering use of UV light to mitigate Mucormycetes spore counts and bacterial agents in northeastern Iran, contributing to the advancement of environmental health and safety practices in hospital settings.

## Introduction

The air in hospital environments contains a wide variety of microbial contaminants, and the levels of these contaminants vary from one department to another within a hospital^1^. In the United States, approximately 2 million people suffer from hospital-acquired infections, and 90,000 people die from these infections every year^2^. Different diseases can be caused by pathogenic infections that are transmitted through the air, posing a significant threat to human health and life^3^. Ventilation, heating, and air conditioning systems can contribute rather to the spread of microorganisms that induce infections^4^. Coughing and talking for five minutes can produce 3000 droplets, while sneezing can produce 40,000 droplets^5^. Sizes larger than 1 µm and smaller than 50 µm are typical for bioaerosols^6^. Biological particles in the air have an aerodynamic diameter that ranges from 0.001 to 100 µm^7^. Inhalation is one of the primary routes through which people are exposed to bacterial, and it can have several adverse health effects, such as cancer, allergies, acute toxicity, and respiratory disorders^8^. Bioaerosols, which can have harmful health effects, are exposed to hospital employees, patients, and visitors^9,10^. A collection of filamentous molds from the orders Mucorales and Entomophthorales are the source of the infection known as mucormycosis. Mucorals can be found in soil, rotting food, bread, dust, and other environmental habitats^11^. Mucormycosis infections can be caused by contact with the skin or open wounds, ingestion of contaminated food, or inhalation of spores in the respiratory system^12^. In affluent nations, mucormycosis mostly affects individuals with severe immunodeficiencies^13^. However, patients with uncontrolled diabetes mellitus or individuals who have had an injury account for a significant portion of mucormycosis cases in impoverished nations^14^. Mucomycosis has a strong tendency to affect blood vessels, leading to thrombosis, necrosis, and tissue infarction, all of which can be fatal^15^. Numerous taxa, including *Mucor*, *Rhizopus, Lichtheimia* (formerly *Absidia*), *Rhizomucor*, *Cunninghamella*, *Apophysomyces*, and *Saksenaea*, are members of the order Mucorales. *Mucor*, *Lichtheimia*, and *Rhizopus* are the three genera most commonly associated with Mucormycosis^16^. Other genera are typically less prevalent. According to research, the most lethal Mucormycetes species in humans are those belonging to the genus *Cunninghamella*^17^. Providing medical services to patients is the fundamental objective of hospital administrators^18^. The hospital should be cleaned up first, though Engineering control measures should be used to reduce the risk of infection^19^. Filtration or dilution, in which fresh ventilation air is introduced into the space to replace any contaminated air, is a traditional remedy^20^. There is a significant demand for efficient indoor air disinfection solutions due to the harmful effects that exposure to airborne infections can have on human health^21^. Strong germicidal effects are known to be caused by ultraviolet germicidal radiation (UVGI), particularly at wavelengths between 180 and 280 nm^22^ . Studies on the effectiveness of conventional UVC in hospital room air disinfection, however, have only been published occasionally^23^. The following describes how UV radiation reduces microorganisms: Microorganisms are exposed to ultraviolet radiation, which penetrates their cell walls and affects nucleic acids and other essential cellular substances, resulting in damage and destruction of cells^24^. Several studies have investigated the effect of photolysis on the removal of microorganisms and found that UV radiation does not significantly affect the destruction of fungi spores, but is effective in eradicating bacterial. Among them, in the study of Maleki and Nazari (2018), which was conducted at Imam Hossein Hospital in Tehran. The ICU department has the highest rate of bacterial contamination. The most abundant bacterial observed were *Enterococcus*, *Pseudomonas* species, coagulase negative *Staphylococcus*, *Klebsiella* species and group D non-enterococcal streptococcus respectively^25^. Also, in the study by Mokhtari et al. It was observed in the burn department, operating theater and emergency department. Fungal contamination was more in the skin section than in other sampling sites. The dominant genus of Gram-positive bacterial was *Staphylococcus epidermidis* and the dominant species of Gram-negative bacterial was *Citrobacter freundii*. The most fungal genera isolated from hospital air samples were *Penicillium*, *Aspergillus niger* and *Aspergillus flavus*^26^. However, a limited number of studies specifically investigate the presence of Mucormycetes and other microorganisms in hospital settings. Therefore, there is an urgent need for more research in this field, especially considering the various conditions and environmental factors that may be associated with the occurrence of Mucormycetes in the air. In this study, our aim is to investigate the presence of Mucormycetes in the indoor air of Mashhad educational, research and therapeutic hospital. In addition, we will assess the density and variety of other airborne microbial agents. By conducting this comprehensive investigation, we hope to gain valuable insights into the prevalence and distribution of Mucormycetes and other microorganisms in the hospital environment. Such information will be crucial to implement effective measures to control and prevent the spread of these potentially harmful pathogens.

## Materials and methods

### Sampling sites

**Figure 1:**
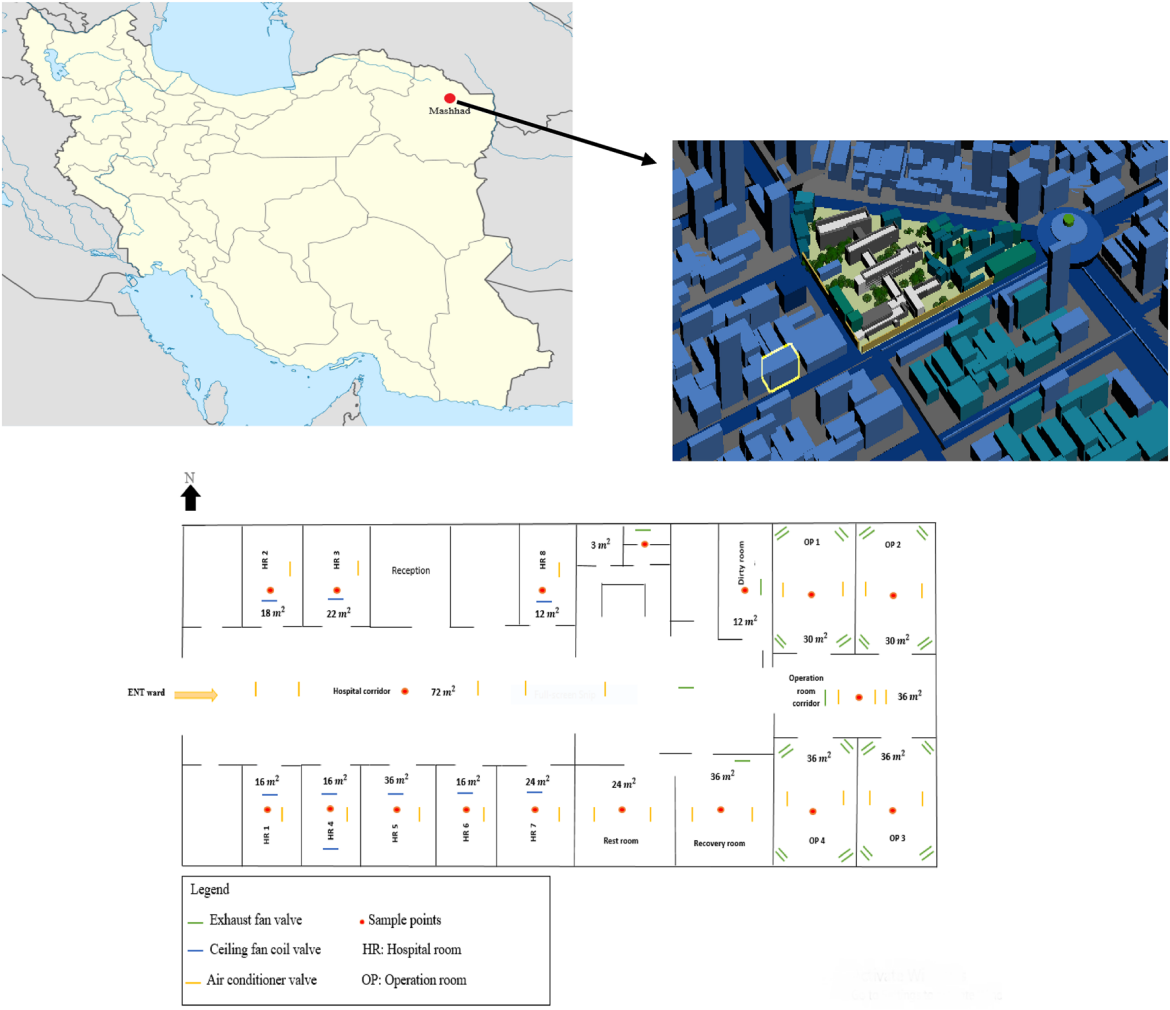
Sampling site^27–29^.

The sampling site for this study was located in Mashhad, which serves as the capital of Razavi Khorasan Province and ranks as the second-largest city in Iran. The local climate is characterized as cold and semi-arid, featuring chilly winters and moderate summers. Mashhad encompasses an area of approximately 280 square kilometers and boasts a population of 3,131,586 people, as per the latest census data. This cross-sectional descriptive study was carried out at the Specialized Teaching and Research Hospital in the year 2021. Presently, the Mashhad University of Medical Sciences operates a sizable and state-of-the-art medical facility with 848 operational beds. This medical center extends its services to patients not only from Mashhad but also from neighboring provinces. Within the hospital, the Ear, Nose, and Throat (ENT) section comprises 30 active beds, encompassing 23 beds designated for adult patients and 7 beds allocated for pediatric patients. The bed occupancy rate in this section stands at an impressive 87%. Given the frequent performance of reconstructive surgeries and examinations conducted under anesthesia, it became imperative to assess the pathogenic factors impacting both patients and the hospital staff. For reference, Fig. 1 provides a schematic representation of the sampling site^27–29^.

### Sample size determination and sampling method

**Figure 2:**
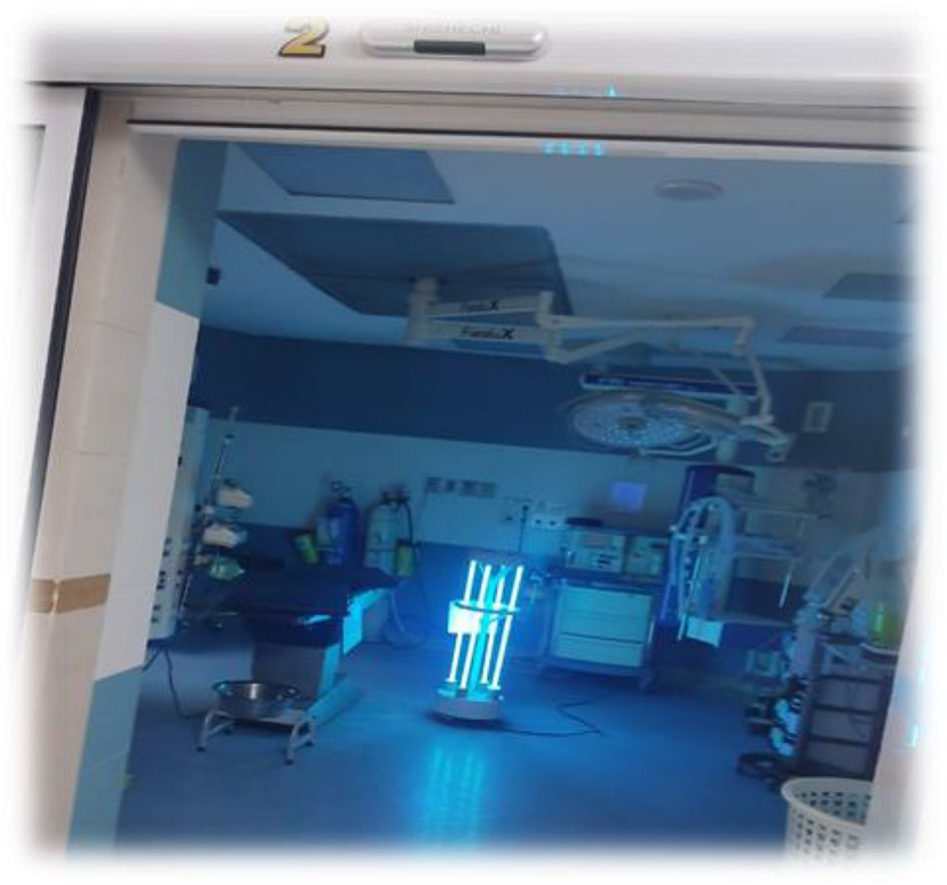
UV C radiation device inside the operating room of the hospital.

In this study, we employed American-made Biostage, following the NIOSH standard method, and utilized Anderson single-stage sampling (active sampling) with a calibrated American-made SKC pump, as outlined by Yang et al.^30^. Air samples were collected for a duration of 90 min, maintaining a flow rate of 15 L per minute, within specialized controlled environments for culturing^31^. The sampling process encompassed various areas within the hospital, including the operating room corridor, washroom, clean room, restroom, and recovery room. Specifically, sampling was carried out in the inpatient section, comprising 8 rooms and a connecting hallway. Systems exhaust fan, ceiling fan and air conditioner have been used for the operating room section and systems fan, ceiling fan and air conditioner for air conditioning the hospital room section. Additionally, we conducted outdoor air sampling for Mucormycetes from two distinct locations surrounding the hospital premises. Overall, a total of 298 samples were gathered, comprising 108 samples for bacterial and 190 samples for fungi analysis. These samples were collected both before and after the implementation of the UV device. The main components of the UV disinfection system include the reactor, lamps, lamp power supply and ballast, a mechanical system to maintain the lamps and the control system. The device had 5 UV lamps, with each lamp emitting 30 watts of radiation and a luminous flux of 1900 lumens (Fig. 2). The operating room space was 30 square meters. The UV disinfection device was activated for 30 min. For the ENT section, the sampling site configuration is depicted in Fig. 1. Notably, the sampling apparatus and connections underwent rigorous cleaning and drying using 70% alcohol before each sample collection to eliminate any potential initial contamination. The sampling device was positioned at a height corresponding to the breathing level of patients and was positioned more than one meter away from walls and other obstructions to ensure accurate data acquisition^32^.

### Enumeration and computation of bacterial and fungal agents

The gathered Bacterial samples underwent incubation within a controlled temperature range of 35 to 37 degrees Celsius. After a 48-h incubation period for water-based culture media, the detection of fungi colonies, such as Cladosporium and Penicillium, usually occurred within a temperature range of 25 °C over a period of 5 to 6 days. There might be slight variations in the preferred temperature ranges for growth and colony formation among different species within these genera. Following the incubation period of 5 to 6 days for fungi culture media, the culture plates were examined, and the resulting colonies were quantified.

### Enumeration and computation of mucormycosis conidia

Active sampling of conidia from both indoor and outdoor environments, utilizing a pump, was conducted on Dichloran Rose Bengal Chloramphenicol (DRBC) culture media and Blood agar. Blood agar as the culture medium for sampling bacterial bioaerosols, supplemented with the antifungal compound Nystatin. DRBC agar is used for environmental monitoring and assessment of fungal contamination in various samples. Subsequently, the collected samples were transported to a dedicated laboratory for Mucormycetes identification, employing standardized protocols. To estimate colony-forming units (CFU), we applied a modification of the Peto and Powell protocol^33^.

## Ethical considerations

The authors affirm that they have fully adhered to ethical considerations, encompassing aspects such as plagiarism, informed consent, research misconduct, data fabrication or falsification, double publication or submission, redundancy, and other related concerns.

## Results and discussion

The spore concentration of Mucormycetes and Bacterial agents in the Ear, Nose, and Throat (ENT) department was evaluated before the implementation of UVC rays. As indicated in Table 1, approximately 56% of the samples yielded positive results.

**Table 1:** Positive cases of ENT ward Mucormycetes samples before and after using UV rays.

Sampling location	Number of positive samples (%) before using UV rays	Number of positive samples (%) after using UV rays	Sampling location	Number of positive samples (%) before using UV rays	Number of positive samples (%) after using UV rays
Operation room1	4 (80%)	3 (60%)	Hospital room 2	1 (20%)	1 (20%)
Operation room2	3 (60%)	2 (40%)	Hospital room 3	3(60%)	2 (40%)
Operation room3	4 (80%)	3 (60%)	Hospital room 4	1 (20%)	0
Operation room4	5 (100%)	3 (60%)	Hospital room 5	3(60%)	3 (60%)
Operation room corridor	5 (100%)	4 (80%)	Hospital room 6	0	2 (40%)
dirty room	3(60%)	2 (40%)	Hospital room 7	2 (40%)	3 (60%)
Rest room	3(60%)	2 (40%)	Hospital room 8	1 (20%)	2 (40%)
Recovery room	2 (40%)	3 (60%)	Hospital corridor	3(60%)	2 (40%)
W.C	3(60%)	3 (60%)	Outdoor1	4 (80%)	2 (40%)
Hospital room 1	3(60%)	2 (40%)	Outdoor2	3(60%)	2 (40%)
Total(average) before using UV rays	56(56%)				
Total(average) after using UV rays	46(46%)				

Table 2 outlines the prevalence of Mucormycetes and bacteria agents in the ear, nose, and throat (ENT) section before exposure to UV rays. The findings reveal that the highest concentration of Mucormycetes was discovered in operating room 1 within the hospital, while the greatest distribution was observed in the outdoor area. Concerning bacteria agents, the highest distribution was noticed in operation rooms 3 and 4, as well as the dirty room.

**Table 2:** Frequency distribution of the spore load of Mucormycetes and bacterial agents in the ENT ward before and after the use of UV rays.

		Operation room1	Operation room 2	Operation room3	Operation room4	Operation room corridor	dirty room	Rest room	Recovery room	W.C	Hospital room 1
Before the use of UV rays	Mucor( mean ± SD)	26.4 ± 25.28	12.8 ± 17.22	20.2 ± 16.24	24.2 ± 16.75	22.8 ± 15.15	20 ± 26.75	17 ± 20.63	10 ± 14.34	19.8 ± 19.37	13.8 ± 14.74
	Bacteria ( mean ± SD)	29.03 ± 9.9	86.55 ± 17.5	57.14 ± 11.5	78.56 ± 14.4	144.8 ± 46.7	124.74 ± 27.8	106.12 ± 28.45	132.25 ± 36.3	114.13 ± 26.7	273.36 ± 41.3
	Fungi ( mean ± SD)	3.44 ± 1.4	3.54 ± 1.4	3.57 ± 1.5	3.22 ± 2.24	29.03 ± 7.3	21.35 ± 6.3	12.9 ± 3.4	16.12 ± 10.5	55.27 ± 15.5	43.75 ± 7.3
After the use of UV rays	Mucor( mean ± SD)	4.6 ± 3.85	4.4 ± 6.19	3.6 ± 3.58	6.8 ± 7.85	5.2 ± 4.44	1 ± 1.41	3 ± 4.24	2.6 ± 2.79	4.4 ± 4.98	3 ± 4.24
	Bacteria ( mean ± SD)	16.58 ± 4.79	35.98 ± 8.43	26.55 ± 4.86	22.83 ± 5.49	54.71 ± 6.73	37.77 ± 16.58	19.50 ± 3.74	88.10 ± 8.26	92.97 ± 9.01	34.51 ± 6.20
	Fungi ( mean ± SD)	0.75 ± 0.66	1.54 ± 0.85	0.38 ± 0.65	1.18 ± 1.05	4.19 ± 2.14	2.71 ± 2.59	6.59 ± 2.6	2.45 ± 4.25	9.39 ± 3.72	12.05 ± 2.04

		Hospital room 2	Hospital room 3	Hospital room 4	Hospital room 5	Hospital room 6	Hospital room 7	Hospital room 8	Hospital corridor	Outdoor1	Outdoor2
Before the use of UV rays	Mucor (mean ± SD)	5.8 ± 12.97	4.6 ± 4.56	2 ± 4.47	7.2 ± 6.91	0	6.4 ± 10.83	4.8 ± 10.73	13.2 ± 18.94	60.8 ± 48.59	57 ± 69.08
	Bacteria (mean ± SD)	238.51 ± 32	138.29 ± 38.39	256.83 ± 27.5	359.37 ± 68.5	136.14 ± 26.6	293.02 ± 67.2	101.66 ± 19.6	234.48 ± 83.9	–	–
	Fungi (mean ± SD)	64.96 ± 12.4	27.03 ± 14.5	115.62 ± 16.5	135.57 ± 27.3	41.93 ± 11.3	117.24 ± 27.6	43.33 ± 9.5	64.96 ± 12.4	–	–
After the use of UV rays	Mucor (mean ± SD)	1.2 ± 2.68	4 ± 5.52	0	4.2 ± 4.27	2.2 ± 3.49	4.2 ± 4.27	2.6 ± 3.97	2.8 ± 4.76	–	–
	Bacteria (mean ± SD)	46.66 ± 5.06	131.12 ± 28.55	61.80 ± 14.44	114.60 ± 6.97	154.98 ± 28.35	75.39 ± 9.24	35.22 ± 8.79	69.79 ± 22.25	–	–
	Fungi (mean ± SD)	22.01 ± 3.26	5.71 ± 3.43	43.92 ± 6.50	35.87 ± 25.57	10.84 ± 9.61	29.14 ± 12.27	12.53 ± 11.40	10.19 ± 9.07	–	–

According to Table 3, about 46% of the samples tested positive after exposure to UV rays. Additionally, the average count of Mucormycetes in the ENT ward, post-UV ray treatment, ranged from 0 to 7.85 ± 6.8. The bacteria load varied between 4.79 ± 16.58 and 28.35 ± 154.98, while the fungi load ranged from 0.65 ± 0.38 to 43.92 ± 6.50. These variations indicated a significant decrease (Table 1).

**Table 3:** Frequency of bacterial and fungal species in ENT before using UV rays.

	Bacteria	Frequency (%)	Fungi	Frequency (%)
1	*Staphylococcus aureus*	23.83	*Penicillium*	28.63
2	*Streptococci*	19.43	*Aspergillus fumigatus*	23.15
3	*Micrococcus*	12.86	*Aspergillus flavus*	19.35
4	*Bacillus*	11.23	*Cladosporium*	12.92
5	*Corynebacterium*	9.05	*Aspergillus niger*	6.30
6	*Acinetobacter*	7.14	*Candida*	4.21
7	*Pseudomonas aeruginosa*	5.92	*Rhizopus*	3.69
8	*Neisseria*	4.25	NO Others	1.74
9	*Escherichia coli*	3.95		
10	NO Others	2.33		

Table 2 illustrates the prevalence of Mucormycetes and bacteria agents in the ear, nose, and throat (ENT) section after the utilization of UV rays. The results showcased a notable reduction in the occurrence of Mucormycetes and fungal agents. Moreover, bacteria agents exhibited decreased levels with various fluctuations.

The average count of Mucormycetes in the ENT ward and the external environment ranged from 0 to 26.4 ± 25.28 and 69.08 ± 59 to 60.8 ± 48.59, respectively. The results of the bioaerosol density measurements are categorized by bioaerosol type and specific areas (Table 2). According to Table 2, Inpatient Room 5 exhibited the highest bacterial contamination load, registering a density of 359.37 ± 68.5. Conversely, Operating Room 1 demonstrated the lowest bacterial contamination load, with a density of 29.03 ± 9.9. Additionally, Inpatient Room 7 exhibited the highest fungi contamination level, with a density of 117.24 ± 27.6, whereas Operating Room Number 4 had the lowest fungal contamination level, measuring 3.22 ± 2.24 in density.

This finding enabled researchers to investigate the effect of UVA radiation on bacterial removal^34^. The results of a study assessing the inactivation of gram-negative and gram-positive bacterial using fluorescent light revealed that ultraviolet radiation (4.28 mW/cm^2^) significantly affects the inactivation of *Bacillus subtilis*. The rate of bacterial destruction for this organism was found to be 0.1279 per minute *Aspergillus* and *Penicillium* can be largely eliminated and destroyed without the application of UVA^35^. This research evaluated the difference in Aspergillus and Penicillium concentrations after exposure to UVA radiation at 0.85 mW/cm^2^, but no significant results were found. However, in the current investigation, UVC radiation was applied for 15 min, and a significant difference in the concentration of fungi load was observed^36^. Different types, intensities, and durations of radiation may have varying effects on the reduction of bacterial and fungi burden. As is well known, UVC rays have a shorter wavelength and more energy than UVA rays. As a result, it causes more harm than UVA. Therefore, due to their low intensity, UVA rays are effective in killing vegetativecells. However, due to its higher intensity, UVC has a significant impact on spore destruction^37^. *mucormycosis* is an invasive and destructive fungal infection that is widely recognized as posing a severe health risk^38^. It is unknown how this disease actually spreads and which types of fungi cause it on an ecological level. Numerous species of Mucormycetes have been isolated from soil, as reported in a recent study conducted in India^39^. Mucormycosis are known to produce numerous conidia to obtain nutrients, facilitate growth, and spread through the air^40^. The amount of Mucormycetes in both indoor and outdoor air is not well understood based on environmental data^41^. Air samples from outdoor areas in the current investigation revealed extremely high Mucormycetes spore counts, ranging from 69,085.7 to 60,848.59 CFU m^−3^. Although the calculation of the number of Mucormycetes in the ambient air has already been assessed, the present investigation found that the Mucormycetes spores load ranged from 0.73 ± 0.96 to 8.60 ± 5.70 CFU m^−3^. In addition, the limited number of studies that have examined the prevalence of different airborne fungi in the open air in the United States of America, Zagreb, Croatia, Iran, and India have revealed a very low occurrence of Mucormycete^42^. The low *Mucormycosis* load calculated in the current study does not agree with these findings. In the current investigation, we found that the spore burden was greater in the environment outside the hospital. It's interesting to note that the autumn season had a higher spore count.

This might be due to the onset of the growing season near Mashhad, which could lead to the dispersal of Mucormycetes from plant and soil fungi. Although Punjab has similar farming practices, no similar trend was noticed there. Therefore, the validity of this claim is questionable. The findings of sampling can also be influenced by variables such as air speed, ambient temperature, relative humidity, time of day, and activity level. In order to reduce the risk of aerosol-borne infections, it is necessary to implement robust procedures for managing the quality of ventilated air. This is particularly important in hospitals, where the patient population includes a variety of immunocompromised individuals who are at risk of developing invasive fungal infections. Lack of routine cleaning and maintenance could make the department's air conditioning ducts a potential source of disease. High-efficiency particulate air (HEPA) filters and other effective measures for managing nosocomial infections should be used to purify the air. This is particularly important due to the increasing number of reports of nosocomial mucormycosis cases originating from India^43^. The use of both coarse and fine air filters in central air conditioning, along with regular cleaning, can be beneficial. This is because installing and maintaining high-efficiency particulate air filtration systems may not be practical in most hospital environments due to their high cost^44^. In the operating room, we also observed a high count of Mucormycetes, which significantly decreased after the completion of the outdoor construction activities. It is common for hospitals to experience air pollution from fungus conidia during building or remodeling^45^. Therefore, to prevent airborne fungal infections during construction or renovation in hospitals, it is necessary to have a multidisciplinary team, engage in careful planning, and conduct environmental monitoring in high-risk units^46^. This is particularly significant because outbreaks of mucormycosis associated with construction-related fungal infections have already been documented^44^.

## Conclusion

High spore loads of Mucormycetes were found in indoor environments in the current investigation, despite the fact that the majority of mucormycosis cases are reported from the community (outdoors). Therefore, further research focused on mucormycosis in community settings is required to gain a proper understanding of the disease epidemiology. Estimating the airborne spore load can also help predict and control a potential outbreak of mucormycosis in hospitals. The application of UV radiation reduces the spore load of Mucormycetes and other bacteria and fungal agents, according to the data obtained from analyzing the indoor air. The occurrence of various hospital infections in patients referred to and hospitalized in the studied departments not only disrupts the healing process, but also leads to the development of diseases, resulting in exorbitant treatment costs. The occurrence of different hospital-acquired infections in patients admitted to and treated in the examined departments hinders their healing process, facilitates the transmission of diseases, and ultimately increases the cost of healthcare. The importance of improving air microbiology conditions in the departments under study, especially in the hospital's operating rooms, which are particularly sensitive, should be recognized. This includes addressing Mucormycetes and other bacterial and fungal agents. By implementing filters that have high performance and efficient operation, utilizing UV disinfection, and ensuring proper hygiene practices among staff and patients, hospital-acquired infections, including mucormycosis, can be prevented.

## Data Availability

The datasets generated and analyzed during the current study are not publicly available due to confidentiality concerns and institutional regulations. However, reasonable requests for data access may be considered by contacting the corresponding author.

## Abbreviations

DRBC: Dichloran Rose Bengal Chloramphenicol
UVGI: Ultraviolet germicidal radiation
ICU: Intensive Care Unit
ENT: Ear, nose, and throat diseases
GIS: Geographic information system
CFU/m^−^^3^: Colony forming units per cubic meter
HEPA: High-efficiency particulate air
NIOSH: National Institute for Occupational Safety and Health
